# Circulating cytokines in predicting development of severe acute pancreatitis

**DOI:** 10.1186/cc13885

**Published:** 2014-05-21

**Authors:** Anne Nieminen, Mikael Maksimow, Panu Mentula, Lea Kyhälä, Leena Kylänpää, Pauli Puolakkainen, Esko Kemppainen, Heikki Repo, Marko Salmi

**Affiliations:** 1 Department of Surgery, Helsinki University Central Hospital, POB 340, Helsinki 00029 HUS, Finland; 2 MediCity Research Laboratory, University of Turku, Tykistönkatu 6A, 20520 Turku, Finland; 3 Department of Medical Microbiology and Immunology, University of Turku, Kiinamyllynkatu 10, 20520 Turku, Finland; 4 Department of Bacteriology and Immunology, Haartman Institute, University of Helsinki, PO Box 21, Helsinki 00014, Finland; 5 National Institute for Health and Welfare, Turku, Finland

## Abstract

**Introduction:**

Severe acute pancreatitis (AP) is associated with high morbidity and mortality. Early prediction of severe AP is needed to improve patient outcomes. The aim of the present study was to find novel cytokines or combinations of cytokines that can be used for the early identification of patients with AP at risk for severe disease.

**Methods:**

We performed a prospective study of 163 nonconsecutive patients with AP, of whom 25 had severe AP according to the revised Atlanta criteria. Admission serum levels of 48 cytokines and growth factors were determined using Bio-Plex Pro Human Cytokine Assay 21-plex and 27-plex magnetic bead suspension panels. Admission plasma levels of C-reactive protein (CRP), creatinine and calcium were measured for comparison. In subgroup analyses, we assessed the cytokine profiles of patients with severe AP (*n* = 14) who did not have organ dysfunction (OD) upon admission (modified Marshall score <2).

**Results:**

Of 14 cytokines elevated in the severe AP group, interleukin 6 (IL-6) and hepatocyte growth factor (HGF) levels were independent prognostic markers of severe AP. IL-6, HGF and a combination of them predicted severe AP with sensitivities of 56.0%, 60.0% and 72.0%, respectively, and specificities of 90.6%, 92.8% and 89.9%, respectively. The corresponding positive likelihood ratio (LR+) values were 5.9, 8.3 and 7.1, respectively. The predictive values of CRP, creatinine and calcium were comparable to those of the cytokines. In subgroup analyses of patients with severe AP and without OD upon admission, we found that IL-8, HGF and granulocyte colony-stimulating factor (G-CSF) levels predicted the development of severe AP, with G-CSF being the most accurate cytokine at a sensitivity of 35.7%, a specificity of 96.1% and a LR+ of 9.1.

**Conclusions:**

IL-6 and HGF levels upon admission have prognostic value for severe AP which is similar to levels of CRP, creatinine and calcium. Although IL-6 and HGF, as either single or combined markers, were not perfect in identifying patients at risk for severe AP, the possibility that combining them with novel prognostic markers other than cytokines might improve prognostic accuracy needs to be studied. The accuracy of IL-8, HGF and G-CSF levels in predicting severe AP in patients without clinical signs of OD upon admission warrants larger studies.

## Introduction

Acute pancreatitis (AP) is usually a mild disease, resolving within days. Severe AP, as defined according to the revised Atlanta criteria [1], accounts for about 20% of cases. The mortality rate in severe AP ranges from 9% to 24% [2,3] and can be as high as 47% to 69% among patients who develop multiple organ dysfunction syndrome [4,5].

In AP, premature activation of trypsin within pancreatic acinar cells causes pancreatic autodigestion, leading to a local inflammatory process. This process is characterized by the release of pro- and anti-inflammatory cytokines and other inflammatory mediators, which recruit neutrophils, monocytes and lymphocytes into the pancreas. In severe AP, the local inflammatory process is amplified and spreads through the circulation throughout the body, resulting in a systemic inflammatory response [6,7]. Systemic inflammation is thought to contribute to the development of organ dysfunction (OD), which may be transient (<48 hours) or persistent (>48 hours). The latter is associated with a 35% to 50% mortality rate [8,9]. Accurate prediction of persistent OD is needed because these patients will benefit from general supportive care, including early fluid resuscitation, in the ICU [10,11].

Although levels of a variety of cytokines, determined upon admission to the hospital, may predict the course of AP [12-15], no cytokine has proved to be useful enough to be incorporated into routine clinical use. Furthermore, to the best of our knowledge, no attempt has been made to discriminate the predictive power of cytokines in patients with OD upon admission from those who are bound to develop OD during hospitalization.

In this study, we analyzed 48 different cytokines in a total of 25 patients with severe AP to determine, upon admission to hospital, novel markers specific for such patients. We used plasma levels of C-reactive protein (CRP), creatinine and calcium as conventional biomarkers. In further analyses of the data, we excluded patients with modified Marshall scores ≥2, whom, we reasoned, already had OD upon admission, compared with the respective patients with modified Marshall scores <2, who developed OD later during hospitalization.

## Methods

### Patients and definitions

In this prospective study conducted at the Helsinki University Central Hospital between June 2003 and January 2008, we assessed 163 nonconsecutive patients with AP admitted within 72 hours of the onset of symptoms. Patients with the signs of chronic pancreatitis were excluded.

AP was diagnosed if two of the following three features were observed: typical abdominal upper epigastric pain, serum or plasma amylase level at least three times greater than the upper limit of normal and characteristic findings of AP on transabdominal ultrasonography, computed tomography or magnetic resonance imaging. The severity of AP was categorized retrospectively according to the revised Atlanta classification system as mild, moderately severe (local complication or transient OD) or severe (persistent OD) [1]. Two patients with severe AP were referred to Helsinki University Central Hospital because they needed to be admitted to the ICU.

**Figure 1:**
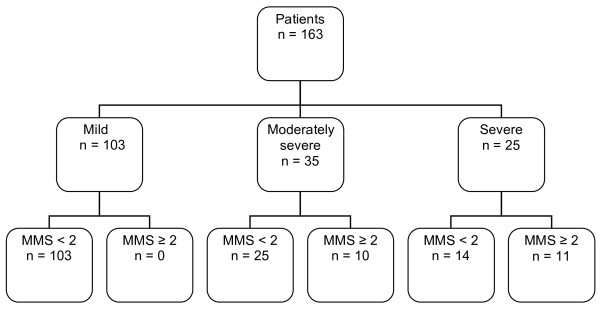
**Patient classifications according to modified Marshall score [**[20]**] and revised Atlanta criteria [**[1]**] upon admission.** MMS, Modified Marshall score.

The Marshall score [16] was calculated according to the method described by Banks *et al*. [1] to assess the presence of OD upon admission. Thus, OD was deemed to be present if the modified Marshall score was ≥2 for one of the three organ systems (respiratory, renal or cardiovascular). A flowchart of the patient distribution is presented in Figure 1.

All patients, or their next of kin, gave their informed consent to participate. The Ethics Committee of the Helsinki University Central Hospital Department of Surgery approved the study.

### Samples and cytokine measurements

Serum samples were collected upon admission to the hospital and stored at -70°C until used for analyses. Each sample (20 μl) was studied by magnetic bead suspension array using the Bio-Plex Pro Human Cytokine 21- and 27-plex panels (Bio-Rad Laboratories, Hercules, CA, USA) according to the manufacturer’s instructions except that the assay reagents were used at half of their recommended concentrations. The 21-plex panel contains interleukin 1α (IL-1α), IL-2 receptor α (IL-2Rα), IL-3, IL-12p40, IL-16, IL-18, cutaneous T-cell attracting chemokine, growth-regulated oncogene α, hepatocyte growth factor (HGF), interferon α2 (IFN-α2), leukemia inhibitory factor (LIF), monocyte chemotactic protein 3 (MCP-3), macrophage colony-stimulating factor (M-CSF), macrophage migration inhibitory factor, monokine induced by IFN-γ, β-nerve growth factor, stem cell factor, stem cell growth factor β, stromal cell–derived factor 1α, tumor necrosis factor β (TNF-β) and TNF-related apoptosis inducing ligand. The 27-plex contains IL-1β, IL-1 receptor antagonist, IL-2, IL-4, IL-5, IL-6, IL-7, IL-8, IL-9, IL-10, IL-12p70, IL-13, IL-15, IL-17A, basic fibroblast growth factor, eotaxin, granulocyte colony-stimulating factor (G-CSF), granulocyte-macrophage colony-stimulating factor (GM-CSF), IFN-γ, IFN-γ-induced protein 10, monocyte chemotactic protein 1 (MCP-1), macrophage inflammatory protein 1α (MIP-1α), MIP-1β, platelet-derived growth factor BB, regulated on activation, normal T cell expressed and secreted (that is, RANTES), TNF-α and vascular endothelial growth factor. The samples were analyzed using the Bio-Plex 200 System, and the results were calculated using Bio-Plex Manager 6.0 software (Bio-Rad Laboratories).

No measurable values for IFN-α2 were obtained from any sample, and thus this cytokine was excluded from data analyses. Also, the majority of the values for LIF, eotaxin, GM-CSF, IL-1α**,** IL-3**,** IL-12p40, IL-15 and TNF-β were below the detectable limit, and therefore these cytokines were scored on a dichotomous scale of detectable or undetectable value. The persons doing the cytokine measurements were unaware of the clinical status of the patients.

Plasma levels of CRP (normal reference range <10 mg/L), creatinine (50 to 90 μmol/L) and calcium (2.15 to 2.51 mmol/L) were determined in accordance with our hospital’s laboratory routine.

### Statistical analysis

Statistical analysis was performed using IBM SPSS version 19 statistical software (SPSS, Chicago, IL, USA). The results are given as medians with ranges and interquartile ranges (IQR). Spearman’s rank correlation was used to test correlations between two continuous variables. For univariate analysis, comparisons between groups were made using the Mann–Whitney *U* test. The level of significance was adjusted using the Bonferroni method by dividing the significance level 0.05 by the number of simultaneous tests. After univariate analysis, parameters were entered into multivariate analysis, and logistic regression analysis was performed to identify independent markers predictive of persistent OD. In the *post hoc* analysis, we determined clinically optimal cutoff values for each cytokine using receiver operating characteristic (ROC) curves, with corresponding sensitivities, specificities, positive likelihood ratios (LR+), negative likelihood ratios (LR-) and diagnostic odd ratios (DORs) with 95% confidence intervals [17]. The DOR is the ratio of the odds of positive test results (OD) among patients with OD to the odds of a positive test result (OD) among the patients without OD. The higher the value, the better the discriminatory test performance is [18]. The clinically optimal cutoff value was defined as the point on the curve where the number or false positives is as low as possible (specificity ≥90%), with the maximum sensitivity, to avoid overtreatment of patients in the ICU. Areas under the ROC curve were also calculated.

## Results

### Patients

The characteristics of the patients are shown in Table 1. The severity of AP was classified as mild in 103 patients (63%), moderately severe in 35 patients (17%) and severe in 25 patients (15%). All of the patients with severe AP developed persistent OD and required invasive mechanical ventilation and/or renal replacement therapy.

**Table 1:** **Characteristics of the patients**^**a**^

**Characteristics**	**All patients (*N *** **= 163)**	**Mild or moderately severe AP (*n *** **= 138)**	***P-*** **value**^**b**^	**Severe AP (*n *** **= 25)**			
				**All (*N *** **= 25)**	**MMS <2 (*n *** **= 14)**	***P-*** **value**	**MMS ≥2 (*n *** **= 11)**
Men	120 (73.6)	96 (69.6)	0.006	24 (96.0)	13 (92.9)	NS	11 (100.0)
Age, years	48 (18 to 87)	49 (18 to 87)	NS	43 (29 to 81)	41 (29 to 81)	NS	51 (29 to 69)
Etiology							
Alcohol	113 (69.3)	91 (65.9)	0.028	22 (88.0)	11 (78.6)	NS	11 (100)
Biliary	35 (21.5)	34 (24.6)	0.021	1 (4.0)	1 (7.1)	NS	0
Other cause	11 (6.7)	9 (6.5)	NS	2 (8.0)	2 (14.3)	NS	0
Idiopathic	4 (2.5)	4 (2.9)	NS	0	0	NS	0
Onset of symptoms, hr	24 (1 to 72)	24 (1 to 72)	NS	24 (3 to 72)	18 (3 to 48)	<0.001	48 (24 to 72)
C-reactive protein, mg/L	19 (3 to 435)	16 (3 to 426)	0.002	94 (3 to 435)	30 (3 to 229)	<0.001	294 (22 to 435)
Creatinine, μmol/L	64 (31 to 1086)	62 (31 to 313)	<0.001	92 (47 to 1086)	68 (47 to 147)	<0.001	248 (92 to 1086)
Calcium, mmol/L	2.2 (1.27 to 2.73)	2.23 (1.27 to 2.73)	<0.001	1.86 (1.39 to 2.30)	2.04 (1.49 to 2.30)	0.013	1.67 (1.39 to 2.17)
Length of hospital stay, days	6 (1 to 93)	5 (1 to 41)	<0.001	26 (1 to 93)	28 (1 to 93)	NS	17 (1 to 49)
Mechanical ventilation	25 (15.3)	2 (1.4)	<0.001	23 (92.0)	13 (92.9)	NS	10 (90.9)
Renal replacement therapy	16 (9.8)	0	<0.001	16 (64.0)	7 (50.0)	NS	9 (81.8)
OD within 24 hours	30 (18.4)	10 (7.2)	<0.001	20 (80.0)	9 (64.3)	NS	11 (100)
OD on days 1 to 7	5 (3.1)	0	<0.001	5 (20.0)	5 (37.5)	NS	0 (0)
Mortality	8 (4.9)	1 (0.7)	0.014	7 (28.0)	2 (14.3)	NS	5 (45.5)

^a^AP, Acute pancreatitis; MMS, Modified Marshall score; NS, Not significant; OD, organ dysfunction. ^b^Significance of the difference between mild or moderately severe AP (*n* = 138) and severe AP (*n* = 25). The severe AP group is categorized into two subgroups: admission modified Marshall Score <2 or ≥2. Data are median (range) or *n* (%).

OD was present upon admission in 21 patients (13%). Ten of these cases were transient OD, which responded rapidly to treatment and resolved within 48 hours. In these cases, the severity of AP was classified as moderately severe. Eleven patients with severe AP had OD upon admission, and another nine patients developed OD within 24 hours after admission. Five patients developed OD within 1 to 7 days after admission. There were no cases of new-onset OD after the first week of treatment.

The overall mortality rate was 4.9%. Seven (28%) of the twenty-five patients with severe AP died. Two of them were not admitted to the ICU because of their old age and comorbidities, and they died within 48 hours. One patient with moderately severe AP died as a result of carbon dioxide retention.

### Cytokine profiles of patients with severe acute pancreatitis

The levels of 14 of 47 cytokines measured were significantly higher in the severe AP group than in the mild and moderately severe AP groups (*P* < 0.001 after Bonferroni adjustment) (Table 2). The corresponding differences in the levels of the other 33 cytokines were not significant (Table 3).

**Table 2:** **The 14 cytokines that differed between mild or moderately severe acute pancreatitis patients and severe acute pancreatitis patients**^**a**^

	**All patients (*N *** **= 163)**			**Admission MMS <2 (*n *** **= 142)**		
**Cytokines**	**Mild or moderately severe AP (*n *** **= 138)**	***P-*** **value**^**b**^	**Severe (*n *** **= 25)**	**Mild or moderately severe AP (*n *** **= 128)**	***P-*** **value**^**b**^	**Severe AP (*n *** **= 14)**
G-CSF	119.7 (66.6-198.2)	<0.001	260.2 (132.5 to 1011.8)	118.41 (66.6 to 174.3)	0.0007	234.0 (151.2 to 531.1)
GRO-α	58.8 (35.7-92.3)	<0.001	128.2 (98.4 to 169.1)	54.5 (33.2 to 82.5)	0.002 (NS)	118.5 (62.4 to 158.4)
HGF	1055.5 (764.7-1730.6)	<0.001	3613.0 (2055.3 to 6348.8)	989.6 (753.6 to 1501.6)	<0.001	2202.6 (1554.3 to 3305.5)
IL-2Rα	214.2 (150.6-316.7)	<0.001	483.3 (385.6 to 673.3)	209.5 (145.5 to 290.4)	0.0015 (NS)	449.7 (228.1 to 504.3)
IL-6	59.7 (15.3-202.1)	<0.001	428.9 (138.5 to 1796.3)	49.9 (14.7 to 185.6)	0.0013 (NS)	234.5 (66.2 to 1931.3)
IL-8	26.6 (19.3-41.9)	<0.001	82.4 (46.4 to 115.6)	25.41 (18.0 to 37.6)	<0.001	59.7 (32.0 to 102.8)
IL-18	139.6 (91.6-187.1)	<0.001	202.8 (151.8 to 305.4)	131.5 (90.7 to 181.5)	0.062 (NS)	162.4 (135.3 to 201.6)
LIF^c^	0 (0)	<0.001	1 (0 to 1)	0 (0)	0.002 (NS)	1 (0 to 1)
M-CSF	14.5 (8.1-28.1)	<0.001	40.2 (18.6 to 68.6)	12.4 (5.5 to 21.5)	0.007 (NS)	28.7 (12.2 to 52.0)
MCP-1	49.0 (21.0-111.2)	<0.001	125.0 (64.6 to 199.6)	47.6 (30.0 to 100.6)	0.032 (NS)	92.1 (36.3 to 185.2)
MCP-3	17.4 (0.57-53.6)	<0.001	54.58 (24.3 to 98.7)	16.3 (0.2 to 47.9)	0.006 (NS)	52.0 (16.5 to 102.1)
β-NGF	5.79 (3.65-9.1)	<0.001	11.3 (7.4 to 15.9)	5.5 (3.4 to 8.5)	0.007 (NS)	10.4 (4.5 to 15.5)
SCF	114.1 (85.2-147.0)	0.0008	155.8 (109.2 to 256.0)	111.6 (82.0 to 143.3)	0.012 (NS)	148.5 (107.3 to 218.9)
SDF-1α	87.6 (53.5-151.5)	<0.001	163.6 (104.4 to 226.3)	86.8 (52.8 to 148.5)	0.010 (NS)	171.3 (85.1 to 234.8)

^a^β-NGF, β-nerve growth factor; G-CSF, granulocyte colony-stimulating factor; GRO-α, Growth-related oncogene α; HGF, Hepatocyte growth factor; IL, Interleukin; IL-1Ra, Interleukin 1 receptor antagonist; IL-2Rα, Interleukin 2 receptor α; SDF, stromal derived factor; LIF, Leukemia inhibitory factor; MCP, Monocyte chemotactic protein; M-CSF, Macrophage colony-stimulating factor; SDF, Stromal derived factor; SCF, Stem cell factor; SDF, Stromal cell–derived factor IQR, interquartile range; NS, not significant. ^b^Level of significance is defined as *P* < 0.001 (Bonferroni-adjusted). ^c^0 = Undetectable value, 1 = Detectable value. Cytokine levels (pg/ml) are expressed as median (IQR).

**Table 3:** **Levels of 33 cytokines showing no significant difference between mild or moderately severe and severe acute pancreatitis**^**a**^

**Cytokines**	**Mild or moderately severe AP (*n *** **= 138)**	***P-*** **value**^**b**^	**Severe AP (*n *** **= 25)**
CTACK	993.1 (746.4 to 1,556.1)	0.033 (NS)	693.3 (652.5 to 1,150.6)
Eotaxin^c^	1 (0 to 1)	0.133 (NS)	1 (0 to 1)
FGF	25.9 (8.6 to 44.6)	0.123 (NS)	19.4 (0.4 to 35.2)
GM-CSF^c^	0 (0)	0.663 (NS)	0 (0 to 1)
IFN-γ	99.5 (50.7 to 151.0)	0.772 (NS)	82.4 (50.9 to 149.5)
IP-10	379.3 (244.5 to 594.0)	0.475 (NS)	446.2 (222.6 to 825.3)
IL-1α^c^	0 (0 to 1)	0.473 (NS)	1 (0 to 1)
IL-1β	1.9 (0.8 to 3.0)	0.961 (NS)	1.9 (0.5 to 3.4)
IL-1Ra	121.1 (64.8 to 260.0)	0.131 (NS)	202.6 (70.9 to 669.8)
IL-2	7.6 (2.2 to 13.4)	0.796 (NS)	6 to 7 (1.7 to 12.9)
IL-3^c^	0 (0)	0.008 (NS)	0 (0 to 1)
IL-4	5.1 (2.6 to 7.5)	0.261 (NS)	3.8 (2.3 to 6.4)
IL-5	2.7 (1.6 to 4.1)	0.091 (NS)	1.8 (1.0 to 3.4)
IL-7	5.9 (3.9 to 10.5)	0.482 (NS)	5.2 (3.9 to 9.4)
IL-9	19.5 (7.3 to 37.8)	0.410 (NS)	16.1 (1.7 to 38.7)
IL-10	8.2 (3.9 to 17.7)	0.012 (NS)	15.8 (6.2 to 42.3)
IL-12p70	18.7 (11.2 to 40.4)	0.442 (NS)	15.5 (9.0 to 36.5)
IL-12p40^c^	0 (0)	0.691 (NS)	0 (0)
IL-13	7.2 (3.9 to 13.5)	0.249 (NS)	6.5 (2.6 to 12.1)
IL-15^c^	0 (0)	0.018 (NS)	0 (0 to 1)
IL-16	220.2 (99.9 to 786.8)	0.120 (NS)	341.3 (157.2 to 980.1)
IL-17A	79.8 (33.2 to 155.9)	0.084 (NS)	45.8 (9.2 to 98.4)
MIP-1α	6.2 (3.7 to 8.0)	0.215 (NS)	4.6 (2.4 to 7.1)
MIP-1β	67.1 (42.6 to 90.2)	0.830 (NS)	63.6 (40.1 to 95.8)
MIF	695.8 (348.5 to 1,553.9)	0.004 (NS)	1461.2 (713.4 to 4,529.4)
MIG	1,415.5 (821.0 to 2,348.2)	0.021 (NS)	2,147.5 (1,257.4 to 4,268.7)
PDGF-BB	1,183.6 (360.4 to 2,475.2)	0.063 (NS)	583.3 (207.8 to 1,427.3)
RANTES	2,905.6 (1,110.3 to 4,511.1)	0.093 (NS)	1,877.2 (787.5 to 3,090.9)
SCGF-β	31452.4 (21610.3 to 46978.0)	0.004 (NS)	47129.9 (30743.1 to 56343.2)
TRAIL	57.4 (33.3 to 92.0)	0.042 (NS)	84.8 (51.8 to 122.7)
TNF-α	20.8 (10.3 to 31.9)	0.730 (NS)	18.2 (9.7 to 31.5)
TNF-β^c^	0 (0 to 1)	0.187 (NS)	0 (0)
VEGF	29.9 (17.7 to 71.1)	0.436 (NS)	23.6 (17.4 to 64.2)

^a^AP, Acute pancreatitis; CTACK, Cutaneous T-cell attracting chemokine; FGF, Basic fibroblast growth factor; GM-CSF, Granulocyte-macrophage colony-stimulating factor; IFN, Interferon; IL, Interleukin; IL-1Ra, Interleukin 1 receptor antagonist; IP-10, Interferon-γ-induced protein 10; VEGF, vascular endothelial growth factor; MIF, Macrophage migration inhibitory factor; MIG, Monokine induced by interferon γ; MIP, Macrophage inflammatory protein; VEGF, vascular endothelial growth factor; PDGF-BB, Platelet-derived growth factor, two BB chains; RANTES, Regulated on activation, normal T cell expressed and secreted; SCGF-β, Stem cell growth factor β; TNF, Tumor necrosis factor; TRAIL, Tumor necrosis factor–related apoptosis-inducing ligand; VEGF, Vascular endothelial growth factor IQR, interquartile range; NS, Not significant. ^b^Level of significance is defined as *P* < 0.001 (Bonferroni-adjusted). ^c^0 = Undetectable value, 1 = Detectable value. Data are median (IQR).

By stepwise forward logistic regression analysis of the 14 cytokines, we identified IL-6 (*P* = 0.006) and HGF (*P* < 0.001) as independent prognostic markers of severe AP. Many of the other 14 cytokines (M-CSF, HGF, IL-8, MCP-1 and G-CSF) had a strong positive Spearman’s correlation with IL-6 (*r* > 0.6) (Table 4). M-CSF, IL-8, MCP-1 and G-CSF did not improve the explanatory power of the regression model and thus were excluded from the stepwise analysis.

**Table 4:** **Cytokines showing strong correlation with interleukin 6**^**a**^

**Cytokines**	**Spearman’s r**	***P-*** **value**
M-CSF	0.62	<0.001
HGF	0.67	<0.001
IL-8	0.67	<0.001
MCP-1	0.71	<0.001
G-CSF	0.72	<0.001

^a^G-CSF, Granulocyte colony-stimulating factor; HGF, Hepatocyte growth factor; IL, Interleukin; MCP, Monocyte chemotactic protein.

IL-6 and HGF levels predicted severe AP with sensitivities of 48.0% and 60.0%, respectively, and specificities of 93.5% and 92.8%, respectively (Table 5). In the combined regression model of IL-6 and HGF, sensitivity was improved to 72.0% and specificity remained high at 89.9%. The LR+ values for IL-6, HGF and the combined model were 7.4, 8.3 and 7.1, respectively, and the DOR values were 13.2, 19.8 and 22.8, respectively. None of the differences between IL-6, HGF and the combined model were statistically significant (Table 5). Additional file 1 shows the diagnostic performance of all of the 14 cytokines that could differentiate mild or moderately severe AP from severe AP.

**Table 5:** **Diagnostic performances of interleukin 6 and hepatocyte growth factor and conventional markers in predicting severe acute pancreatitis in all patients (*N *** **= 163)**^**a**^

**Predictive marker with optimal cutoff point**	**AUC**	**Sensitivity (%)**	**Specificity (%)**	**LR+**	**LR-**	**DOR**
IL-6 (>501.6 pg/ml)	0.81 (0.72 to 0.90)	48.0 (30.0 to 66.5)	93.5 (88.1 to 96.5)	7.4 (3.5 to 15.6)	0.56 (0.38 to 0.81)	13.2 (4.7 to 37.3)
HGF (>3,020.1 pg/ml)	0.87 (0.81 to 0.94)	60.0 (40.7 to 0.77)	92.8 (87.1 to 96.0)	8.3 (4.2 to 16.3)	0.43 (0.27 to 0.70)	19.2 (6.9 to 53.6)
IL-6 + HGF	0.89 (0.82 to 0.96)	72.0 (52.4 to 85.7)	89.9 (83.7 to 93.9)	7.1 (4.1 to 12.3)	0.31 (0.17 to 0.59)	22.8 (8.1 to 64.0)
CRP (>227 mg/L)	0.692 (0.57 to 0.82)	40.0 (23.4 to 59.3)	93.5 (88.0 to 96.5)	6.1 (2.8 to 13.6)	0.64 (0.47 to 0.89)	9.6 (3.4 to 27.2)
Creatinine (>141 μmol/L)	0.76 (0.65 to 0.88)	40.0 (23.4 to 59.3)	96.3 (91.7 to 98.4)	10.9 (4.1 to 29.1)	0.62 (0.45 to 0.86)	17.5 (5.3 to 57.9)
Calcium (<1.91 mmol/L)	0.81 (0.70 to 0.91)	57.9 (36.3 to 76.9)	93.2 (87.6 to 96.4)	8.5 (4.1 to 17.8)	0.45 (0.27 to 0.77)	18.8 (6.0 to 58.4)

^a^IL, interleukin; CRP, C-reactive protein; IL, interleukin; HGF, Hepatocyte growth factor; IL, Interleukin; AUC, Area under curve; DOR, Diagnostic odds ratio; LR-, Negative likelihood ratio; LR+, Positive likelihood ratio; 95% confidence intervals are given in parentheses.

**Figure 2:**
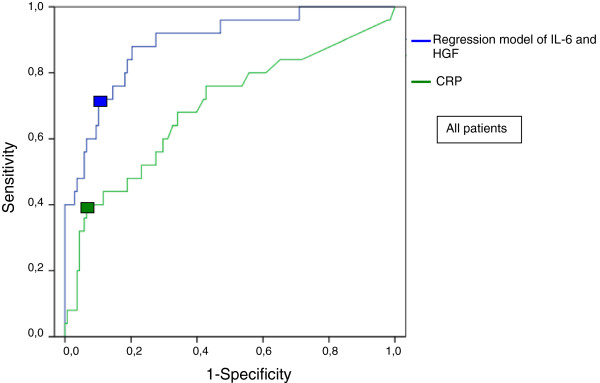
**Receiver operating characteristic curves of C-reactive protein and interleukin 6 plus hepatocyte growth factor for prediction of severe acute pancreatitis in the entire acute pancreatitis patient cohort (*N *= 163).** Boxes indicate clinically optimal cutoff points used to calculate the sensitivity and specificity of each biomarker listed in Table 5. CRP, C-reactive protein; HGF, Hepatocyte growth factor; IL, Interleukin.

The discriminatory power of plasma calcium <1.91 mmol/L, creatinine >141 μmol/L and CRP >227 mg/L was comparable to that of IL-6 and HGF (Table 5). When calcium, creatinine and CRP were added into the logistic regression analysis with IL-6 and HGF, calcium was also able to predict severe AP independently (*P* = 0.023), as were IL-6 (*P* = 0.025) and HGF (*P* = 0.027), but creatinine (*P* = 0.86) and CRP (*P* = 0.45) were not. The ROC curves for the logistic regression model of IL-6 and HGF and that for CRP are presented in Figure 2, and their clinically optimal cutoff points are listed in Table 5.

### Predicting severe acute pancreatitis in patients with modified Marshall score <2 upon admission

In the patients who developed persistent OD after admission to the hospital (*n* = 14), compared with patients who did not (*n* = 128), the levels of IL-8, HGF and G-CSF were significantly higher (*P* < 0.001, Bonferroni-adjusted) (Table 2). We did not perform multivariate analysis in this subgroup, owing to the low number of patients. We therefore determined how many of these 14 OD patients could be identified using the same clinically optimal cutoff points that we used earlier for the whole patient cohort (Table 5). G-CSF correctly predicted severe AP in 5 of 14 patients (Table 6). IL-8 detected one patient, and HGF identified two additional patients. Thus, the combination of these three cytokines detected eight of fourteen OD patients. The diagnostic performance of each cytokine (IL-8, HGF and G-CSF) and that of the combination are presented in Table 7.

**Table 6:** **Performance of biomarkers to predict severe acute pancreatitis in patients with admission modified Marshall Score <2 (*N *** **= 14)**^**a**^

**Severe AP patients**	**IL-8 >88.1 pg/ml**	**HGF >3,020.1 pg/ml**	**G-CSF >477.7 pg/ml**	**CRP >227 mg/L**	**Creatinine >141 μmol/L**	**Calcium <1.91 mmol/L**
1	-	-	-	-	-	-
2	-	**+**	**+**	-	-	**+**
3	-	-	-	-	-	-
4	**+**	-	-	-	-	^b^
5	-	**+**	**+**	-	-	^b^
6	-	**+**	-	**+**	-	^b^
7	-	-	-	-	**+**	^b^
8	-	-	-	-	-	**+**
9	-	-	-	-	-	-
10	**+**	-	**+**	-	-	**+**
11	-	-	-	-	-	-
12	**+**	-	**+**	-	-	-
13	-	**+**	-	-	-	-
14	**+**	-	**+**	-	-	**+**

^a^AP, Acute pancreatitis; CRP, C-reactive protein; G-CSF, Granulocyte colong-stimulating factor; HGF, Hepatocyte growth factor; IL, Interleukin. +, Value above cutoff point (except for calcium below cutoff point). -, Value below cutoff point (except for calcium above cutoff point). ^b^Missing data.

**Table 7:** **Diagnostic performances of biomarkers markers in predicting severe acute pancreatitis in patients with admission modified Marshall score <2 (*N *** **= 142)**^**a**^

**Predictive marker and optimal cutoff point**	**AUC**	**Sensitivity (%)**	**Specificity (%)**	**LR+**	**LR-**	**DOR**
IL-8 (>88.1 pg/ml)	0.79 (0.68 to 0.90)	25.6 (11.7 to 54.7)	95.3 (90.2 to 97.8)	6.1 (2.0 to 19.0)	0.75 (0.54 to 1.05)	8.1 (2.0 to 33.6)
HGF (>3,020.1 pg/ml)	0.83 (0.73 to 0.92)	28.6 (11.7 to 54.7)	95.3 (0.90 to 97.8)	6.1 (2.0 to 19.0)	0.75 (0.54 to 1.05)	8.1 (2.0 to 33.6)
G-CSF (>477.7 pg/ml)	0.78 (0.65 to 0.90)	35.6 (16.3 to 61.2)	96.1 (91.2 to 98.3)	9.1 (3.0 to 27.7)	0.67 (0.45 to 0.99)	13.7 (3.3 to 56.1)
CRP (>227 mg/L)	0.54 (0.37 to 0.70)	7.1 (1.3 to 31.5)	95.3 (90.2 to 97.8)	1.5 (0.2 to 11.8)	0.97 (0.2 to 11.8)	1.6 (0.2 to 14.0)
Creatinine (>141 μmol/L)	0.62 (0.45 to 0.79)	7.1 (1.3 to 31.5)	99.2 (95.7 to 99.9)	9.1 (0.6 to 37.2)	0.94 (0.91 to 1.08)	9.7 (0.57 to 164.2)
Calcium (<1.91 mmol/L)	0.74 (0.60 to 0.88)	36.4 (15.2 to 64.6)	94.3 (88.7 to 97.2)	6.4 (2.2 to 18.5)	0.68 (0.43 to 1.06)	9.5 (2.2 to 40.2)
G-CSF + IL-8 + HGF		57.1 (32.6 to 78.6)	90.6 (84.3 to 94.6)	6.1 (3.0 to 12.3)	0.47 (0.26 to 0.87)	12.9 (3.8 to 43.4)

^a^IL, Interleukin; CRP, C-reactive protein; IL, interleukin; G-CSF, Granulocyte colony-stimulating factor; HGF, Hepatocyte growth factor; IL, Interleukin; AUC, Area under the curve; DOR, Diagnostic odds ratio; LR-, Negative likelihood ratio; LR+, Positive likelihood ratio. 95% confidence intervals are given in parentheses.

**Figure 3:**
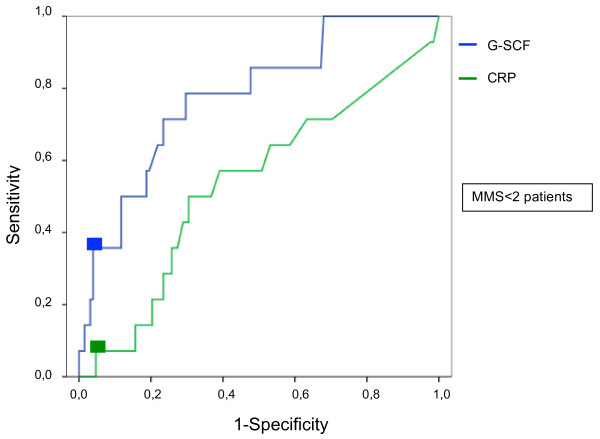
**Receiver operating characteristic curves of granulocyte colony-stimulating factor and C-reactive protein for prediction of severe acute pancreatitis in patients with admission modified Marshall scores <2 (*N *= 142).** Boxes indicate clinically optimal cutoff points used to calculate the sensitivity and specificity of each biomarker given in Table 7. CRP, C-reactive protein; G-CSF, Granulocyte colony-stimulating factor; MMS, Modified Marshall score.

In the subgroup of patients who developed persistent OD after admission, calcium had a sensitivity of 36.4%, a specificity of 94.3%, a LR+ value of 6.4 and a DOR value of 9.5. These values were similar to the respective values for G-CSF (Table 6). The sensitivity of CRP and creatinine each was 7.1%, indicating poor performance in predicting OD. The ROC curves of G-CSF and CRP are presented in Figure 3.

### Predicting severe acute pancreatitis in patients with admission modified Marshall scores ≥2

When comparing the levels of 14 cytokines in the patients with transient OD (*n* = 10) or persistent OD (*n* = 11), we found that the levels of HGF were higher in the patients with persistent OD (*P* = 0.007); however, the difference was not significant after we applied the Bonferroni correction. IL-6 and IL-8 had *P*-values of 0.067 and 0.057, respectively, and the rest of the cytokines had *P*-values >0.1.

## Discussion

The results of our present study show that the levels of 14 of 47 cytokines were significantly higher in the severe AP group than in the patients with mild or moderately severe AP. Regression analysis, however, revealed that only IL-6 and HGF were independent predictive markers of severe AP. Both IL-6 and HGF are multifunctional cytokines that play many roles in inflammation, and the serum levels of these cytokines reflect the magnitude of the inflammatory response.

IL-6 is a proinflammatory cytokine released by macrophages in response to tissue injury, and it stimulates the synthesis of acute phase proteins (for example, CRP) in the liver. HGF stimulates mitogenesis, cell motility and matrix invasion, and thus it has a central role in angiogenesis, tumorigenesis and tissue regeneration [19]. Both IL-6 [12,20] and HGF [21,22] have been demonstrated to have prognostic value for AP upon admission. These studies, together with the results of the present study, indicating LR+ values 7.4 and 8.3 for IL-6 and HGF, respectively, for predicting severe AP, thus give credence to the view etc., thus giving credence to the view that single cytokines such as IL-6 and HGF may not be perfect predictors of severe AP. Our results also show that the LR+ value for the combination of IL-6 and HGF was 7.1, indicating that combining the cytokines did not improve their predictive accuracy for severe AP. One reason for this may be the strong Spearman’s correlation between IL-6 and HGF, and, indeed, with many other cytokines (Table 4). Moreover, the sensitivities, specificities and LR+ were determined using *post hoc* analysis in which the cutoff points were set in the same population. If the analyses had been done prospectively with preset cutoff values, the predictive power of biomarkers would most probably have been even lower. Taken together, the results suggest that none of the cytokines alone or combinations of them would be useful in clinical practice, which would require LR+ values >10.

In agreement with previous studies [23-25], our results show that many severe AP patients already have OD upon presentation. Consequently, to find true prognostic markers, such patients should be excluded from further analysis. We did so in the subgroup analysis with a limited number of severe AP patients (*n* = 14). When we compared the severe AP groups with admission modified Marshall scores <2 or ≥2, we found that the latter group had had symptoms significantly longer and had significantly higher CRP and creatinine levels upon admission, but otherwise no other clinical marker or symptom differed between the groups. In the group of severe AP patients who presented with modified Marshall scores <2, HGF, as well as also IL-8 and G-CSF, measured upon admission predicted later development of OD. The combination of HGF, G-CSF and IL-8 identified 8 of 14 of these severe AP patients. Thus, in 6 of 14 patients, none of these cytokines were raised above the cutoff level, although there was no difference in the clinical course of the disease. The reason for this may derive from variations in immune responses between individuals, the kinetics of the cytokines and/or the phase of AP. This raises the question whether combining cytokines with other, non-cytokine-related prognostic markers might improve the predictive power for severe AP. Several markers, other than cytokines, have been found to be of prognostic value in OD associated with systemic inflammation [26-30]. Furthermore, novel biomarkers will most probably be discovered by means of systems biology, as reviewed by Skibsted *et al*. [31].

The crucial question in clinical practice is how to distinguish, upon admission to the hospital, those patients with transient OD from those with persistent OD. At present, no means of identifying such patients exist. In our present study, the limited number of persistent OD patients had higher levels of HGF, IL-6 and IL-8, but the differences were not statistically significant. Whether these cytokines, as individual or combined markers, can aid in distinguishing between transient and persistent OD needs to be studied in a larger number of OD patients upon admission to the hospital.

Although no specific treatment for AP exists so far, concomitantly with better understanding of the underlying pathophysiology, immunomodulatory treatment has become a matter of interest. However, many patients already have OD upon admission [23]. In addition, AP patients may develop immune suppression rapidly [32]. In such patients, anti-inflammatory therapy may be detrimental. In some patients, immune suppression is confined mainly to the circulation and the inflammatory stage proceeds in other body compartments, such as the lungs [33]. In this complex setting, the determination of immune inflammatory status may aid in the selection of appropriate immune-modulating therapy to prevent or alleviate OD [34].

## Conclusions

We analyzed the levels of 48 cytokines in 163 patients with AP upon admission to the hospital. IL-6 and HGF were independent predictive markers of severe AP; however, neither cytokine nor the combination of them was perfect in identifying AP patients at risk for severe disease. In fact, the results are similar to those for CRP, creatinine and calcium. IL-8, HGF and G-CSF levels could be used to predict severe AP in patients without clinical signs of OD upon admission. To the best of our knowledge, this study is the largest to date with regard to the number of cytokines screened in a group of AP patients. The possibility that combining cytokines with prognostic markers other than cytokines improves the prediction of severe AP warrants further studies.

## Key messages

• The levels of 14/47 cytokines upon admission to the hospital were higher in patients with severe AP than in those with mild or moderately severe AP.

• IL-6 and HGF were independent predictors of severe AP.

• The predictive value of IL-6 and HGF was comparable to that of CRP, creatinine and calcium.

• Many patients who will develop severe AP, did not have signs of OD upon admission to hospital.

• IL-8, HGF and G-CSF is able to predict severe AP in patients without clinical signs of OD at the time of hospital admission.

## Abbreviations

AP: Acute pancreatitis; AUC: Area under the curve; β-NGF: β nerve growth factor; CRP: C-reactive protein; CTACK: Cutaneous T-cell attracting chemokine; DOR: Diagnostic odds ratio; FGF: Basic fibroblast growth factor; G-CSF: Granulocyte colony-stimulating factor; GM-CSF: Granulocyte-macrophage colony-stimulating factor; HGF: Hepatocyte growth factor; IFN: Interferon; IL: Interleukin; IL-1Ra: Interleukin 1 receptor antagonist; IL-2Rα: Interleukin 2 receptor α; IP-10: Interferon-γ-induced protein 10; LIF: Leukemia inhibitory factor; LR: Likelihood ratio; MCP: Monocyte chemotactic protein; M-CSF: Macrophage colony-stimulating factor; MIF: Macrophage migration inhibitory factor; MIG: Monokine induced by interferon γ; MIP: Macrophage inflammatory protein; OD: Organ dysfunction; PDGF-BB: Platelet-derived growth factor, two BB chains; RANTES: Regulated on activation, normal T cell expressed and secreted; ROC: Receiver operating characteristic curve; SCF: Stem cell factor; SCGF-β: Stem cell growth factor β; SDF: Stromal cell–derived factor; TNF: Tumor necrosis factor; TRAIL: Tumor necrosis factor–related apoptosis-inducing ligand; VEGF: Vascular endothelial growth factor.

## Competing interests

The authors declare that they have no competing interests.

## Authors’ contributions

AN and LKyh collected clinical data and participated in data analyses. AN and HR drafted the manuscript. MM analyzed the cytokine levels. PM was a supervisor of the data analyses and participated in designing the study. LKyl, EK and PP participated in designing and coordinating the study and provided supervision. MS and HR designed and supervised the study. All authors critically revised the manuscript and read and approved the final version.

## Supplementary Material

Additional file 1 Diagnostic performances of the 14 cytokines that could differentiate mild or moderately severe AP from severe AP. Click here for file

## References

[B1] Banks PA Bollen TL Dervenis C Gooszen HG Johnson CD Sarr MG Tsiotos GG Vege SS Acute Pancreatitis Classification Working Group Classification of acute pancreatitis—2012: revision of the Atlanta classification and definitions by international consensus Gut 2013 62 102 111 10.1136/gutjnl-2012-302779 23100216

[B2] Halonen KI Leppaniemi AK Puolakkainen PA Lundin JE Kemppainen EA Hietaranta AJ Haapiainen RK Severe acute pancreatitis: prognostic factors in 270 consecutive patients Pancreas 2000 21 266 271 10.1097/00006676-200010000-00008 11039471

[B3] Gloor B Müller CA Worni M Martignoni ME Uhl W Büchler MW Late mortality in patients with severe acute pancreatitis Br J Surg 2001 88 975 979 10.1046/j.0007-1323.2001.01813.x 11442530

[B4] McKay CJ Buter A Natural history of organ failure in acute pancreatitis Pancreatology 2003 3 111 114 10.1159/000070078 12748419

[B5] Vege SS Gardner TB Chari ST Munukuti P Pearson RK Clain JE Petersen BT Baron TH Farnell MB Sarr MG Low mortality and high morbidity in severe acute pancreatitis without organ failure: a case for revising the Atlanta classification to include “moderately severe acute pancreatitis.” Am J Gastroenterol 2009 104 710 715 10.1038/ajg.2008.77 19262525

[B6] Norman J The role of cytokines in the pathogenesis of acute pancreatitis Am J Surg 1998 175 76 83 10.1016/S0002-9610(97)00240-7 9445247

[B7] Kylänpää ML Repo H Puolakkainen PA Inflammation and immunosuppression in severe acute pancreatitis World J Gastroenterol 2010 16 2867 2872 10.3748/wjg.v16.i23.2867 20556831 PMC2887581

[B8] Buter A Imrie CW Carter CR Evans S McKay CJ Dynamic nature of early organ dysfunction determines outcome in acute pancreatitis Br J Surg 2002 89 298 302 10.1046/j.0007-1323.2001.02025.x 11872053

[B9] Johnson CD Abu-Hilal M Persistent organ failure during the first week as a marker of fatal outcome in acute pancreatitis Gut 2004 53 1340 1344 10.1136/gut.2004.039883 15306596 PMC1774183

[B10] Banks PA Freeman ML Practice guidelines in acute pancreatitis Am J Gastroenterol 2006 101 2379 2400 10.1111/j.1572-0241.2006.00856.x 17032204

[B11] Forsmark CE Baillie J AGA Institute technical review on acute pancreatitis Gastroenterology 2007 132 2022 2044 10.1053/j.gastro.2007.03.065 17484894

[B12] Leser HG Gross V Scheibenbogen C Heinisch A Salm R Lausen M Rückauer K Andreesen R Farthmann EH Schölmerich J Elevation of serum interleukin-6 concentration precedes acute-phase response and reflects severity in acute pancreatitis Gastroenterology 1991 101 782 785 1907253 10.1016/0016-5085(91)90539-w

[B13] Brivet FG Emilie D Galanaud P Pro- and anti-inflammatory cytokines during acute severe pancreatitis: an early and sustained response, although unpredictable of death Crit Care Med 1999 27 749 755 10.1097/00003246-199904000-00029 10321665

[B14] Chen CC Wang SS Lee FY Chang FY Lee SD Proinflammatory cytokines in early assessment of the prognosis of acute pancreatitis Am J Gastroenterol 1999 94 213 218 10.1111/j.1572-0241.1999.00709.x 9934758

[B15] Mayer J Rau B Gansauge F Beger HG Inflammatory mediators in human acute pancreatitis: clinical and pathophysiological implications Gut 2000 47 546 552 10.1136/gut.47.4.546 10986216 PMC1728074

[B16] Marshall JC Cook DJ Christou NV Bernard GR Sprung CL Sibbald WJ Multiple organ dysfunction score: a reliable descriptor of a complex clinical outcome Crit Care Med 1995 23 1638 1652 10.1097/00003246-199510000-00007 7587228

[B17] Newcombe RG Two-sided confidence intervals for the single proportion: comparison of seven methods Stat Med 1998 17 857 872 10.1002/(SICI)1097-0258(19980430)17:8<857::AID-SIM777>3.0.CO;2-E 9595616

[B18] Glas AS Lijmer JG Prins MH Bonsel GJ Bossuyt PM The diagnostic odds ratio: a single indicator of test performance J Clin Epidemiol 2003 56 1129 1135 10.1016/S0895-4356(03)00177-X 14615004

[B19] Nakamura T Nishizawa T Hagiya M Seki T Shimonishi M Sugimura A Tashiro K Shimizu S Molecular cloning and expression of human hepatocyte growth factor Nature 1989 342 440 443 10.1038/342440a0 2531289

[B20] Aoun E Chen J Reighard D Gleeson FC Whitcomb DC Papachristou GI Diagnostic accuracy of interleukin-6 and interleukin-8 in predicting severe acute pancreatitis: a meta-analysis Pancreatology 2009 9 777 785 10.1159/000214191 20110745

[B21] Ueda T Takeyama Y Toyokawa A Kishida S Yamamoto M Saitoh Y Significant elevation of serum human hepatocyte growth factor levels in patients with acute pancreatitis Pancreas 1996 12 76 83 10.1097/00006676-199601000-00010 8927623

[B22] Espinosa L Linares PM Bejerano A Lopez C Sanchez A Moreno-Otero R Gisbert JP Soluble angiogenic factors in patients with acute pancreatitis J Clin Gastroenterol 2011 45 630 637 10.1097/MCG.0b013e31820d3533 21750433

[B23] Johnson CD Kingsnorth AN Imrie CW McMahon MJ Neoptolemos JP McKay C Toh SK Skaife P Leeder PC Wilson P Larvin M Curtis LD Double blind, randomised, placebo controlled study of a platelet activating factor antagonist, lexipafant, in the treatment and prevention of organ failure in predicted severe acute pancreatitis Gut 2001 48 62 69 10.1136/gut.48.1.62 11115824 PMC1728186

[B24] Harrison DA D’Amico G Singer M Case mix, outcome, and activity for admissions to UK critical care units with severe acute pancreatitis: a secondary analysis of the ICNARC Case Mix Programme Database Crit Care 2007 11 S1 18275590 10.1186/cc5682 PMC3226113

[B25] Wu BU Conwell DL Update in acute pancreatitis Curr Gastroenterol Rep 2010 12 83 90 10.1007/s11894-010-0091-6 20424979

[B26] Oiva J Mustonen H Kylänpää ML Kyhälä L Kuuliala K Siitonen S Kemppainen E Puolakkainen P Repo H Acute pancreatitis with organ dysfunction associates with abnormal blood lymphocyte signaling: controlled laboratory study Crit Care 2010 14 R207 10.1186/cc9329 21087472 PMC3220021

[B27] Chakraborty S Kaur S Muddana V Sharma N Wittel UA Papachristou GI Whitcomb D Brand RE Batra SK Elevated serum neutrophil gelatinase-associated lipocalin is an early predictor of severity and outcome in acute pancreatitis Am J Gastroenterol 2010 105 2050 2059 10.1038/ajg.2010.23 20179686 PMC2898914

[B28] Yilmaz G Köksal I Karahan SC Mentese A The diagnostic and prognostic significance of soluble urokinase plasminogen activator receptor in systemic inflammatory response syndrome Clin Biochem 2011 44 1227 1230 10.1016/j.clinbiochem.2011.07.006 21816136

[B29] Moore DJ Greystoke A Butt F Wurthner J Growcott J Hughes A Dive C A pilot study assessing the prognostic value of CK18 and nDNA biomarkers in severe sepsis patients Clin Drug Investig 2012 32 179 187 10.2165/11598610-000000000-00000 22217154

[B30] Oiva J Mustonen H Kylänpää ML Kuuliala K Siitonen S Kemppainen E Puolakkainen P Repo H Patients with acute pancreatitis complicated by organ dysfunction show abnormal peripheral blood polymorphonuclear leukocyte signaling Pancreatology 2013 13 118 124 10.1016/j.pan.2013.01.010 23561969

[B31] Skibsted S Bhasin MK Aird WC Shapiro NI Bench-to-bedside review: future novel diagnostics for sepsis—a systems biology approach Crit Care 2013 17 231 10.1186/cc12693 24093155 PMC4057467

[B32] Kylänpää-Bäck ML Takala A Kemppainen E Puolakkainen P Kautiainen H Jansson SE Haapiainen R Repo H Cellular markers of systemic inflammation and immune suppression in patients with organ failure due to severe acute pancreatitis Scand J Gastroenterol 2001 36 1100 1107 10.1080/003655201750422738 11589386

[B33] Cavaillon JM Annane D Compartmentalization of the inflammatory response in sepsis and SIRS J Endotoxin Res 2006 12 151 170 10.1179/096805106X102246 16719987

[B34] Caldwell CC Hotchkiss RS The first step in utilizing immune-modulating therapies: immune status determination Crit Care 2011 15 108 10.1186/cc9397 21349138 PMC3222027

