# Supplementary material for: Circulating cytokines in predicting development of severe acute pancreatitis
Source: Crit Care. 2014 May 21;18(3):R104. doi: 10.1186/cc13885 (PMC4095695; doi:10.1186/cc13885)
Supplement: Additional file 1 — Diagnostic performances of the 14 cytokines that could differentiate mild or moderately severe AP from severe AP. [file cc13885-S1.pdf]

Additional file 1. Diagnostic performances of the fourteen cytokines differing between mild or moderately severe AP and severe AP.

Predictive marker with optimal cut off point	AUC	Sensitivity (%)	Specificity (%)	+LR	-LR	DOR
G-CSF (>477.7 pg/ml)	0.76 (0.66-0.86)	40.0 (23.4-59.3)	93.5 (88.1-96.5)	6.1 (2.8-13.6)	0.64 (0.47-0.89)	9.6 (3.4-27.2)
GROα (>140.0 pg/ml)	0.81 (0.72-0.91)	44.0 (26.7-62.9)	92.0 (86.3-95.5)	5.5 (2.7-11.3)	0.61 (0.43-0.86)	9.1 (3.3-24.7)
HGF (>3020.1 pg/ml)	0.87 (0.81-0.94)	60.0 (40.7-0.77)	92.8 (87.1-96.0)	8.3 (4.2-16.3)	0.43 (0.27-0.70)	19.2 (6.9-53.6)
IL-2Rα (>567.6 pg/ml)	0.81 (0.72-0.90)	32.0 (17.2-51.6)	90.6 (84.6-94.4)	3.4 (1.6-7.3)	0.75 (0.57-0.99)	4.5 (1.6-12.5)
IL-6 (>501.6 pg/ml)	0.81 (0.72-0.90)	48.0 (30.0-66.5)	93.5 (88.1-96.5)	7.4 (3.5-15.6)	0.56 (0.38-0.81)	13.2 (4.7-37.3)
IL-8 (>88.1 pg/ml)	0.85 (0.77-0.92)	48.0 (30.0-66.5)	93.5 (88.1-96.5)	7.4 (3.5-15.6)	0.56 (0.38-0.81)	13.2 (4.7-37.3)
IL-18 (<312.3 pg/ml)	0.74 (0.64-0.84)	24.0 (11.5-43.4)	92.0 (0.86-0.95)	3.0 (1.2-7.4)	0.83 (0.66-1.0)	3.6 (1.2-11.0)
LIF (0/1)[a]	0.67 (0.55-0.79)	56.0 (37.1-73.3)	90.8 (84.2-94.8)	6.1 (3.1-11.7)	0.49 (0.31-0.76)	12.5 (4.6-34.1)
M-CSF (>40.8 pg/ml)	0.79 (0.68-0.89)	48.0 (30.0-66.5)	90.6 (84.6-94.4)	5.1 (2.6-9.8)	0.57 (0.39-0.84)	8.9 (3.4-23.4)
MCP-1 (>189.5 pg/ml)	0.73 (0.63-0.83)	28.0 (14.3-47.6)	90.6 (84.6-94.4)	3.0 (1.3-6.7)	0.80 (0.62-1.0)	3.7 (1.3-10.6)
MCP-3 (>91.2 pg/ml)	0.72 (0.62-0.81)	32.0 (17.2-51.6)	90.6 (84.6-94.4)	3.4 (1.6-7.3)	0.75 (0.57-0.99)	4.5 (1.6-12.5)
β-NGF (>14.6 pg/ml)	0.74 (0.63-0.85)	32.0 (17.2-51.6)	92.8 (87.2-96.0)	4.4 (1.9-10.1)	0.73 (0.56-0.96)	6.0 (2.1-17.4)
SCF (>199.0 pg/ml)	0.71 (0.60-0.83)	36.0 (20.0-55.5)	91.3 (0.85-0.95)	4.1 (2.0-8.8)	0.70 (0.52-0.95)	5.9 (2.2-16.2)
SDF-1α (>214.8 pg/ml)	0.72 (0.62-0.83)	32.0 (17.2-51.6)	94.2 (89.0-97.0)	5.5 (2.3-13.3)	0.72 (0.55-0.95)	7.6 (2.5-23.0)

AUC, Area under curve; +LR, Positive likelihood ratio; -LR, Negative likelihood ratio; DOR, Diagnostic odds ratio; 95 % confidence intervals are given in parentheses.

[a] 0=undetectable value, 1=detectable value
